# Fish Diversity and Environmental Relationships in the Jinsha River During the Initial Phases of the 10‐Year Fishing Ban: A Metabarcoding Approach

**DOI:** 10.1002/ece3.72002

**Published:** 2025-08-14

**Authors:** Yan Zhao, Zhongyuan Wang, Feifei Hu, Zhibin Guo, Jinling Gong, Xuemei Li, Deguo Yang, Tingbing Zhu

**Affiliations:** ^1^ Key Laboratory of Freshwater Biodiversity Conservation, Ministry of Agriculture and Rural Affairs of China, Yangtze River Fisheries Research Institute Chinese Academy of Fisheries Science Wuhan China; ^2^ Freshwater Fisheries Research Center Chinese Academy of Fishery Sciences Wuxi China

**Keywords:** 10‐year fishing ban, eDNA, environmental factors, fish diversity, Jinsha river

## Abstract

Fish diversity is essential for maintaining the balance of aquatic ecosystems, particularly in rivers impacted by overfishing and hydropower projects, such as the Jinsha river, the upstream segment of the Yangtze river. During initial phases (August and November, 2023) of the 10‐year fishing ban in the Yangtze river basin, we investigated fish diversity, seasonal variations, and their correlation with environmental factors in the Jinsha river using environmental DNA (eDNA) metabarcoding. Utilizing two pairs of 12S rRNA primers, MiFish‐U and AcMDB07, we identified 61 fish species across 5 orders, 17 families, and 52 genera, including 4 national protected and 7 invasive alien fish. Among them, Cypriniformes constituted the predominant group within the fish community, accounting for 65.6%. This finding aligns with the results from a recent fish catch study, which recorded 68 species of fish belonging to 4 orders, 15 families, and 48 genera, including 4 national protected species and 8 invasive alien fish. The alpha diversity analysis revealed compositional differences in the fish community across various regions and seasons. Furthermore, key environmental factors, such as water temperature, dissolved oxygen, nitrate nitrogen, total suspended solids, and conductivity, were found to be highly correlated with fish diversity in the Jinsha river. Consequently, we provided detailed seasonal data on fish diversity and its correlations with environmental factors, which will aid in the systematic management and restoration of fisheries and the assessment of the 10‐year fishing ban in the Jinsha river.

## Graphical abstract

Using eDNA metabarcoding, we identified 61 fish species in the Jinsha river during the early stage of the Yangtze river fishing ban. Fish diversity showed seasonal and regional variation, with Cypriniformes as the dominant group. Key environmental factors like temperature and dissolved oxygen were closely linked to diversity patterns.

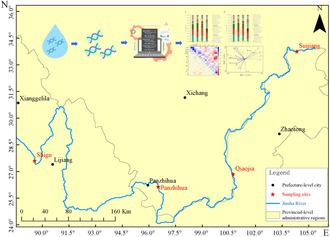

## 1 Introduction

Fish communities, as apex consumers in freshwater ecosystems, serve as vital ecological indicators reflecting the overall balance and stability of these environments. Therefore, fish diversity has played a crucial role in maintaining the health and integrity of aquatic ecosystems (Magurran et al. 2011). However, in recent years, various anthropogenic factors—such as overfishing, captive breeding, water pollution, the invasion of non‐native species, and the construction of hydropower stations and dams—have led to the degradation and loss of fish habitats, resulting in a sharp decline in fish diversity across the Yangtze river basin (Qian et al. 2023). This decline is particularly noticeable in the dramatic reduction of rare and endemic fish populations (Esguícero and Arcifa 2010; Gao et al. 2013; Zhang et al. 2018). Consequently, the erosion of fish diversity in the Yangtze river basin threatens the ecological integrity and long‐term sustainability of freshwater ecosystems. Furthermore, the decline in native fish populations has disrupted the food web, adversely affected other aquatic species, and undermined the overall health of these ecosystems.

The Jinsha river basin, located in the upper reaches of the Yangtze river, is ecologically rich in biodiversity and abundant in fish resources. Based on historical survey data from the past 15 years, the number of fish species in the main stream of the Jinsha river ranges from 60 to 98 (Yang et al. 2014, 2023; Shao et al. 2017; Wang et al. 2024). Among these, 15 species are nationally protected, including *Myxocyprinus asiaticus* , *Procypris rabaudi* , *Onychostom simus*, *Schizothorax davidi* , *Schizothorax chongi* , *Leptobotia elongata* , *Coreius guichenoti* , and others. Additionally, there are approximately 10 invasive alien species, with *Oreochromis niloticus* and *Coptodon zillii* being the most predominant. It also possesses significant potential as a hydropower source. As China's largest hydropower base, the Jinsha river basin serves as a critical source of runoff and nutrients for the Yangtze river. However, with nearly 40 cascade dams either constructed or planned, the basin's fish habitats—both native and non‐native—have been significantly altered or even severely damaged (Sun et al. 2020). Additionally, the construction of these cascade hydropower stations has led to a continuous decline in the concentrations and fluxes of total phosphorus (TP), dissolved total carbon (DTC), and total silicon in the reservoirs, potentially destabilizing river ecosystems and disrupting the food chain (Zhao et al. 2024). As a result, fish community structures in the upper reaches of the Jinsha river have experienced moderate to severe disturbances, characterized by an increase in exotic species and a trend toward smaller sizes and younger age classes (Yan et al. 2022). However, the removal of a low‐head dam and the installation of a fish passage in a tributary of the Jinsha river have significantly increased upstream fish abundance and species richness, helping to reduce disparities between upstream and downstream fish communities (Tan et al. 2024). In 2020, the Yangtze river Basin implemented a 10‐year fishing ban to protect the biodiversity and natural habitats of rare and endemic fish species (Wx 2022). As a result, continuous, long‐term surveys are now essential for assessing the variation, recovery, and diversity of fishery resources in this region.

Traditional fishing survey methods, such as multi‐layer gillnets and ground cages, have several limitations, including gear selectivity, physical damage to specimens, and high labor intensity. Additionally, certain species may be underestimated or entirely missed. While environmental DNA (eDNA) metabarcoding has emerged as a powerful method that integrates traditional ecological techniques with next‐generation sequencing, enabling the detection of species from a single water sample, including those that are otherwise difficult to identify through conventional methods such as microscopy (Deiner et al. 2017; Hanfling et al. 2016), fish eDNA metabarcoding involves the analysis of DNA fragments released into the water by fish throughout their life cycle, which may originate from cell shedding, excrement, germ cells, or decomposed tissues following death (Jerde et al. 2019). This technique can detect trace amounts of DNA, allowing for the identification of invasive and elusive species (Thomsen et al. 2012). For example, eDNA has been successfully employed to track the invasive aquatic plant *Elodea canadensis* in Norway, with changes in DNA concentration closely aligning with the plant's growth cycle (Angles d'Auriac et al. 2019). Additionally, Balasingham et al. detected three at‐risk species—Eastern Sand Darter ( *Ammocrypta pellucida* ), Northern Madtom ( *Noturus stigmosus* ), and Silver Shiner ( *Notropis photogenis* )—alongside the invasive Round Goby ( *Neogobius melanostomus* ) in two tributaries of the Great Lakes in Ontario, Canada (Balasingham et al. 2018). As a rapidly emerging tool, eDNA is increasingly utilized to monitor multiple species across diverse ecosystems, including freshwater environments, over varying temporal scales.

The structure and diversity of fish communities are intricately linked to the ecological characteristics of their habitats, exhibiting distinct response patterns to environmental variables (Heino et al. 2015). Key environmental factors influencing species diversity and abundance include altitude, water temperature, stream width and depth, pH, and turbidity (Yan et al. 2022). Furthermore, various other factors—such as chlorophyll‐a, ammonium nitrogen (NH3—N), dissolved oxygen (DO), total nitrogen (TN), chemical oxygen demand (COD), and altitude—collectively impact the α‐diversity of fish populations in the Yangtze river (Qian et al. 2023). Additionally, Cheng et al. demonstrated that, alongside temperature and DO, reservoir age significantly influences fish community diversity in the Wujiang river, a tributary of the Yangtze river (Cheng et al. 2024). Similarly, Shen et al. found that fish communities in the tributaries of the upper Yangtze river were more strongly influenced by key environmental factors—such as water temperature, DO, electrical conductivity, and ammonia nitrogen—than those in the mainstem (Shen et al. 2024). The heterogeneity of these environmental factors across spatiotemporal scales plays a crucial role in shaping the dynamics of fish composition and community structure, thereby influencing biodiversity patterns and species interactions within aquatic ecosystems. This variability leads to fluctuations in species abundance, distribution, and community composition, which are essential for understanding ecological processes and managing aquatic habitats.

Current research on fish diversity in the Jinsha river is impeded by fragmented data and inadequate species identification, particularly concerning rare and endangered species. In the summer (August 2023) and autumn (November 2023), eDNA metabarcoding technology was utilized to assess fish diversity at four sites along the river, while also investigating the relationships between fish communities and environmental factors. The findings may offer valuable insights for future ecological research and conservation initiatives in the Jinsha river.

## 2 Materials and Methods

### 2.1 Study Area and eDNA Metabarcoding Sampling

**FIGURE 1:**
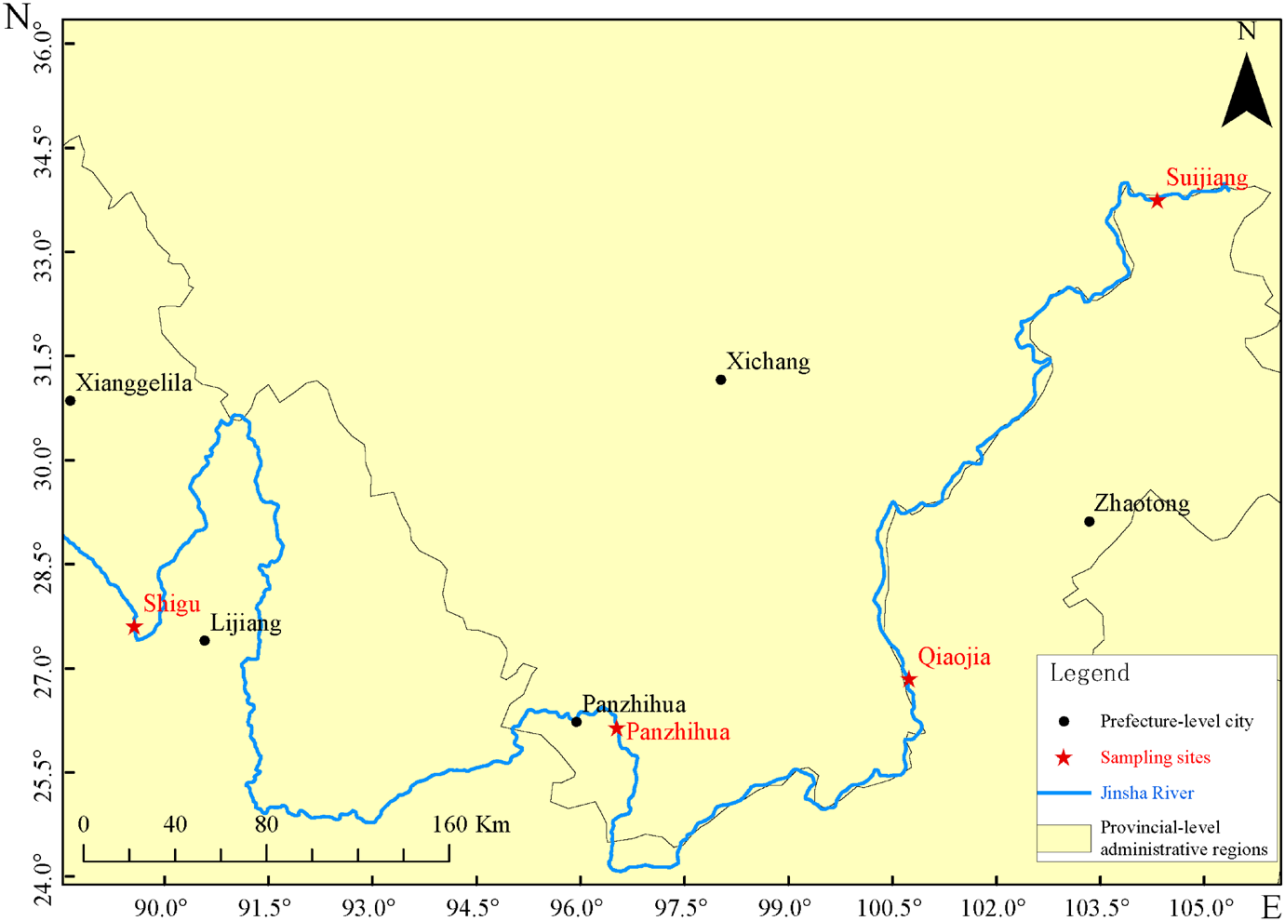
eDNA sampling site map of Jinsha river mainstream.

Four sampling sites along the main stream of the Jinsha river were systematically selected for this study: Shigu (SG), Panzhihua (PZH), Qiaojia (QJ) and Suijiang (SJ) (Figure 1). Water samples were collected during two sampling periods: summer (August 2023) and autumn (November 2023). At each site, a total of 6 L of water was collected, with 2 L per sample obtained during three separate collection events. A plexiglass water collector was used to gather the samples. Prior to sampling, all equipment—including the water collector and storage containers—was disinfected by soaking in 10% sodium hypochlorite for 30 min, followed by thorough rinsing three times with on‐site environmental water. During the collection process, disposable, sterilized latex gloves were worn and replaced immediately between samples to prevent contamination. To further monitor and control contamination, filtration blanks (sterile water filtered using the same setup) were processed alongside environmental samples to detect any potential contamination introduced during field handling or filtration. The collected water samples were stored on ice and filtered within 6 h of collection using a vacuum suction pump and a 0.45 μm pore size mixed cellulose membrane (Whatman, UK). Filtration equipment was thoroughly cleaned between samples using 10% bleach and rinsed with sterile water. After filtration, the filter membranes were transferred into sterile tubes, rapidly frozen in liquid nitrogen, and stored at −80°C until DNA extraction. The study was conducted with approval from the relevant fishery authorities and adhered to the 10‐year fishing ban implemented in the Yangtze river basin.

### 2.2 DNA Extraction and PCR Amplification

DNA was extracted from the filtration membranes using the Qiagen DNeasy Blood&Tissue Kit (Germany) according to the manufacturer's instructions. The concentration and quality of the extracted DNA were assessed using a NanoDrop 2000 spectrophotometer and 1% agarose gel electrophoresis, respectively. A blank filter membrane moistened with distilled water was used as a negative control to minimize potential contamination and experimental errors. Two primer pairs, MiFish‐U and AcMDB07, targeting the 12S rRNA region (Bylemans et al. 2018; Miya et al. 2015), were selected for amplification (Table 1). The total PCR reaction volume was 50 μL, consisting of 25 μL Taq DNA polymerase, 2 μL of each forward and reverse primer (10 μM), 5 μL DNA template, and 16 μL ddH_2_O. The PCR cycling conditions were as follows: initial denaturation at 95°C for 5 min; 37 cycles of denaturation at 95°C for 30 s, annealing at 54°C for 45 s, and extension at 72°C for 40 s; followed by a final extension at 72°C for 7 min and storage at 4°C. After amplification, the PCR products were analyzed using 2% agarose gel electrophoresis. Mixed PCR products were then purified through gel extraction using the OMEGA Gel Extraction Kit (USA). Target DNA fragments were eluted with TE buffer. The purified products were subsequently sent to Guangdong Meige Gene Technology Co. Ltd. for high‐throughput sequencing.

**TABLE 1:** eDNA primer.

Primer	Sequence	Length (bp)	References
MiFish‐U	F: GTCGGTAAAACTCGTGCCAGC R: CATAGTGGGGTATCTAATCCCAGTTTG	180	(Miya et al. 2015)
AcMDB07	F: GCCTATACCGCCGTCG R: GTACACTTACCATGTTACGACTT	300	(Bylemans et al. 2018)

### 2.3 Data Processing and Statistical Analyses

The raw sequencing data obtained from high‐throughput sequencing were subjected to initial quality control using Fastp (v0.12.4), which included adapter trimming and removal of low‐quality reads (Chen et al. 2018). The quality of the reads before and after filtering was assessed using FastQC (v0.11.9). The filtered paired‐end reads were subsequently merged using Usearch (v11.0.667), with primer sequences removed based on alignment to both ends of the reads (Edgar 2010). Merged reads shorter than 150 bp were discarded. All merged reads from individual samples were pooled, and VSEARCH (v2.15.2) was used to dereplicate the sequences and remove those with an abundance of fewer than four reads. Operational taxonomic units (OTUs) were clustered at 98% sequence similarity. Chimeric sequences were identified and removed using the UCHIME3 algorithm, resulting in a final OTU sequence set.

The merged sample reads were mapped to the OTU sequence set to generate the final OTU table. Representative OTU sequences were then aligned against reference sequences for the 12S rRNA gene metabarcoding region, including databases such as MitoFish, NCBI, and BOLD, using BLAST (v2.2.31) (Sato et al. 2018). Sequences with alignment lengths shorter than 90% were discarded. Taxonomic assignments were made based on a similarity threshold of ≥ 98% (McClenaghan et al. 2020). Additionally, OTUs assigned to the same taxonomic unit were merged, and non‐fish sequences were excluded from the dataset.

### 2.4 Environmental Factors and Diversity Indices

In this study, physical environmental factors, including water temperature (WT), pH, turbidity (NTU), conductivity (EC), and dissolved oxygen (DO), were measured in situ using a HACH HQ40D multiparameter water quality analyzer (USA) in accordance with a former study (Meng et al. 2023). Additionally, nutrient factors such as total nitrogen (TN), ammonium nitrogen (NH_4_^+^—N), nitrate nitrogen (NO_3_^−^—N), nitrite nitrogen (NO_2_^−^—N), total phosphate (TP), phosphate (PO_4_^3−^—P) and total suspended solids (TSS) were conducted with a portable multi‐parameter spectrophotometer (Hach DR1990, Hach, USA) according to previous studies (Meng et al. 2022, 2025). The Chao1, Shannon, Pielou, Maragalef, and Simpson diversity indices were used to assess the alpha diversity of the fish community structure. Principal Coordinate Analysis (PCoA) was performed using the vegan package (version 2.4.3) in R (version 3.3.1) to visualize community composition patterns. To further explore differences among groups, Permutational Multivariate Analysis of Variance (PERMANOVA) was conducted. The coefficient of determination (*R* ^2^) and statistical significance (*p* < 0.05) were used to interpret seasonal variation in fish community structure. Additionally, Spearman correlation analysis was applied to investigate the relationships between fish diversity indices and environmental factors in the mainstream of the Jinsha river.

## 3 Results

### 3.1 Fish Species Composition by eDNA Metabarcoding

**FIGURE 2:**
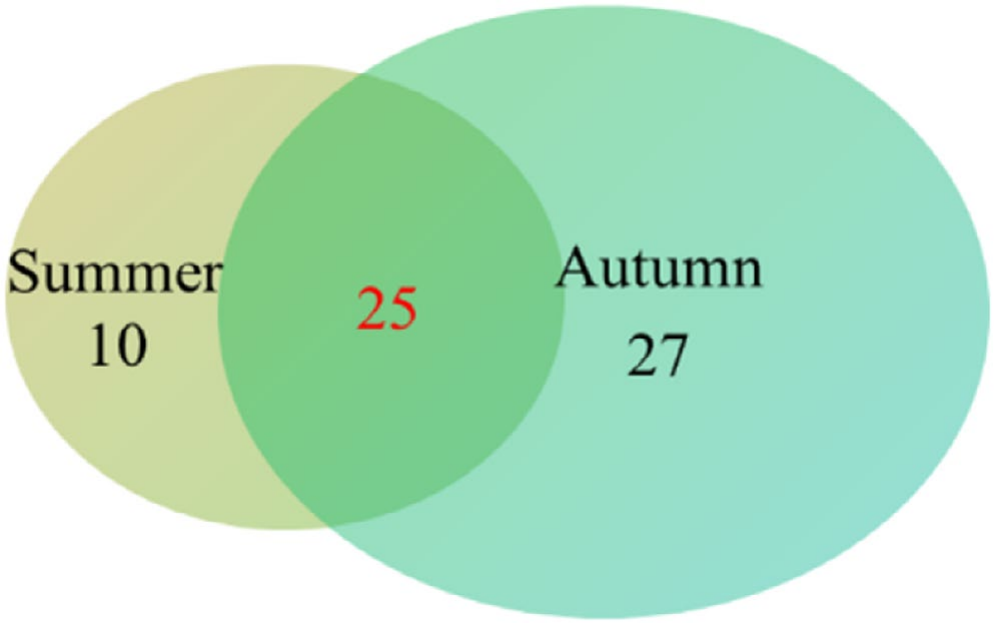
Comparison of fish species detected in the Jinsha river during summer and autumn.

A total of 878 OTUs were obtained, and taxonomic annotation was performed on the raw data. After excluding non‐fish species and marine fish, 61 freshwater fish species were identified in the mainstem of the Jinsha river using eDNA metabarcoding. These species belonged to 52 genera, 17 families, and 5 orders. The order Cypriniformes dominated the fish community, comprising 40 species from 35 genera and 4 families, accounting for 65.6% of the total species. This group included 34 species from the family Cyprinidae, 3 from Cobitidae, 2 from Balitoridae, and 1 from Catostomidae. The second most abundant group was Siluriformes, which included 10 species from 9 genera and 6 families, representing 16.4% of the total species. Among these were 3 species from Sisoridae and Bagridae, as well as 1 species each from Loricariidae, Ictaluridae, Amblycipitidae, and Siluridae. Perciformes accounted for 14.8%, comprising 9 species from 6 genera and 5 families, including 3 species from Gobiidae, 3 from Cichlidae, and 1 species each from Percidae, Centrarchidae, and Channidae. Both Salmoniformes and Synbranchiformes were represented by 1 species each, together contributing 3.2% of the total (Table 2). Among the identified species, four are listed as nationally protected: *Myxocyprinus asiaticus* , *Liobagrus kingi* , *Procypris rabaudi* , and *Euchiloglanis kishinouyei*. Seasonally, 35 species were detected in summer, distributed across 4 orders, 11 families, and 28 genera. In contrast, 52 species were identified in autumn, covering 5 orders, 16 families, and 46 genera. A total of 25 species were shared between the two seasons (Figure 2).

**TABLE 2:** List of fish species detected in the Jinsha river by eDNA metabarcoding.

Order	Family	Species	SG	PZH	QJ	SJ
Cypriniformes	Cyprinidae	*Acanthorhodeus chankaensis*	√	√	√	√
		*Ancherythroculter kurematsui*			√	√
		*Carassius auratus*	√	√	√	√
		*Ctenopharyngodon idella*	√	√	√	√
		*Culter alburnus*	√	√	√	√
		*Cyprinus carpio*	√	√	√	√
		*Pseudorasbora parva*	√	√	√	√
		*Hemiculter leucisculus*	√	√	√	√
		*Hemiculter tchangi*	√	√	√	√
		*Hemiculter bleekeri*	√			
		*Rhodeus sinensis*	√	√	√	√
		*Saurogobio dabryi*	√	√	√	√
		*Xenocypris argentea*	√	√	√	√
		*Zacco platypus*	√	√	√	√
		*Acrossocheilus yunnanensis*	√	√	√	√
		*Hypophthalmichthys molitrix*	√	√	√	√
		*Aristichys nobilis*	√	√	√	√
		*Schizothorax kozlovi*	√	√	√	√
		*Abbottina obtusirostris*	√	√	√	√
		*Abbottina rivularis*	√	√	√	√
		*Anabarilius liui*	√	√	√	√
		*Ochetobius elongatus*	√	√	√	√
		*Coreius heterodon*				√
		*Megalobrama amblycephala*	√	√	√	√
		*Procypris rabaudi*	√	√		
		*Opsariichthys bidens*				√
		*Pseudolaubuca engraulis*	√	√	√	√
		*Ptychobarbus kaznakovi*	√	√	√	√
		*Rhinogobio typus*	√	√	√	√
		*Sinibrama taeniatus*	√	√	√	√
		*Spinibarbus sinensis*	√	√	√	√
		*Schizopygopsis malacanthus*	√	√	√	√
		*Rhynchocypris lagowskii*	√	√	√	√
		*Myxocyprinus asiaticus*		√		√
	Catostomidae	*Schistura fasciolata*	√	√	√	√
	Cobitidae	*Triplophysa orientalis*	√	√	√	√
		*Triplophysa stenura*	√	√	√	√
		*Misgurnus anguillicaudatus*	√	√	√	√
	Balitoridae	*Jinshaia sinensis*	√	√	√	√
		*Jinshaia abbreviata*	√	√	√	√
Perciformes	Centrarchidae	*Micropterus salmoides*				√
	Channidae	*Channa gachua*	√	√	√	√
	Cichlidae	*Coptodon zillii*	√	√	√	√
		*Oreochromis aureus*			√	√
		*Oreochromis niloticus*	√	√	√	√
	Gobiidae	*Rhinogobius cliffordpopei*	√	√	√	√
		*Rhinogobius giurinus*	√	√	√	√
		*Rhinogobius brunneus*	√	√	√	√
	Percidae	*Sander lucioperca*	√	√	√	√
Siluriformes	Sisoridae	*Euchiloglanis kishinouyei*	√		√	√
		*Pareuchiloglanis anteanalis*	√	√	√	√
		*Glyptothorax sinensis*		√		
	Siluridae	*Silurus asotus*	√	√	√	√
	Bagridae	*Tachysurus fulvidraco*	√	√	√	√
		*Tachysurus vachellii*	√	√	√	√
		*Pseudobagrus pratti*	√	√	√	√
	Loricariidae	*Pterygoplichthys pardalis*	√	√	√	√
	Ictaluridae	*Ictalurus punctatus*	√	√	√	√
	Amblycipitidae	*Liobagrus kingi*	√	√	√	√
Salmoniformes	Salangidae	*Neosalanx taihuensis*	√	√	√	√
Synbranchiformes	Synbranchidae	*Monopterus albus*	√	√	√	√

*Note:* √ indicates detection of the species.

Abbreviations: PZH, Panzhihua; QJ, Qiaojia; SG, Shigu; SJ, Suijiang.

An analysis of species composition across the four sampling sites revealed that all locations consistently detected 52 fish species. These included *Acanthorhodeus chankaensis*, *Carassius auratus* , *Ctenopharyngodon idella* , *Culter alburnus* , *Cyprinus carpio* , *Pseudorasbora parva* , *Hemiculter leucisculus* , and *Hemiculter tchangi* , resulting in an overall detection rate of 85.2%. Additionally, seven invasive alien species were identified through eDNA analysis, namely *Rhynchocypris lagowskii*, *Micropterus salmoides* , *Coptodon zillii*, *Oreochromis aureus* , *Oreochromis niloticus* , *Sander lucioperca* , and *Pterygoplichthys pardalis* .

### 3.2 Relative Sequence Abundance Analyses

**FIGURE 3:**
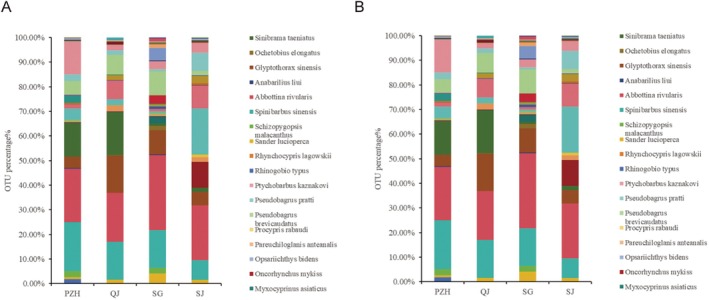
Relative abundance of fish genus (A) and species (B) (OTU similarity is greater than 98%) in each sampling site of the Jinsha river.

Further analysis of relative sequence abundance revealed variations in fish community composition at both the genus and species levels across the four sampling sites (Figure 3A,B). The top 20 most abundant genera were identified at each site (Figure 3A). Among these, *Ctenopharyngodon* and *Coptodon* were consistently detected across all four locations, with *Ctenopharyngodon* showing the highest relative abundance, reaching up to 65%. At the genus level, the five most dominant genera at each site were as follows: in PZH, *Ctenopharyngodon*, *Coptodon*, *Hemiculter*, *Hypophthalmichthys*, and *Rhinogobius*; in QJ, *Schizothorax*, *Hemiculter*, *Ctenopharyngodon*, *Coptodon*, and *Cyprinus*; in SG, *Ctenopharyngodon*, *Coptodon*, *Schizothorax*, *Hemiculter*, and *Cyprinus*; in SJ, *Rhinogobius*, *Ctenopharyngodon*, *Hypophthalmichthys*, *Neosalanx*, and *Coptodon*. At the species level, the top 20 most abundant species were also recorded at each site (Figure 3B). Notably, *Ctenopharyngodon idella* and *Coptodon zillii* consistently ranked among the top five species across all locations. At the PZH site, the five most abundant species were *Ctenopharyngodon idella* , *Coptodon zillii*, *Hemiculter leucisculus* , *Aristichthys nobilis*, and *Hemiculter tchangi* . The QJ site exhibited a similar composition, with *Ctenopharyngodon idella* , *Coptodon zillii*, *Hemiculter leucisculus* , *Hemiculter tchangi* , and *Cyprinus carpio* . At the SG station, the top five species were *Ctenopharyngodon idella* , *Coptodon zillii*, *Cyprinus carpio* , *Hemiculter tchangi* , and *Triplophysa orientalis* . Similarly, at the SJ site, the dominant species were *Ctenopharyngodon idella* , *Rhinogobius cliffordpopei* , *Neosalanx taihuensis* , *Rhinogobius giurinus* , and *Coptodon zillii*.

Overall, based on relative abundance across all sites, the dominant fish species identified through eDNA monitoring were *Ctenopharyngodon idella* , *Coptodon zillii*, *Hemiculter leucisculus* , *Aristichthys nobilis*, *Hemiculter tchangi* , *Cyprinus carpio* , *Rhinogobius cliffordpopei* , and *Neosalanx taihuensis* .

### 3.3 Fish Species Coverage Rate Based on eDNA

Previous studies using conventional fishing methods, including gillnets and cast nets, have documented between 60 and 98 fish species in the mainstream of Jinsha river (Shao et al. 2017; Wang et al. 2024; Yang et al. 2023, 2014) (Table 3). In the present study, conducted from 2022 to 2023, similar gear‐based sampling was performed, yielding a total of 68 fish species, which were classified into 4 orders, 15 families, and 48 genera. Among these, the order *Cypriniformes* was predominant, comprising 51 species and accounting for 75.0% of the total species richness. Additionally, five species were identified as nationally protected, and seven species were classified as non‐native (Wang et al. 2024). A comparative analysis of conventional capture methods and eDNA metabarcoding revealed a total of 91 fish species detected by the two approaches combined, belonging to 5 orders, 19 families, and 62 genera. The eDNA approach detected 61 species, slightly fewer than those recorded by conventional fishing. A total of 38 species were commonly detected by both methods, whereas eDNA metabarcoding uniquely identified 23 species that were not recorded by traditional capture‐based surveys (Table 4).

**TABLE 3:** Historical investigation results of fish species in the Jinsha river.

Year	Area	Number of fish species	References
2009–2012	Panzhihua Yalong river estuary to Geliping river section	60	(Yang et al. 2014)
2013–2017	Panzhihua section	65	(Shao et al. 2017)
2017–2021	Mainstream of Jinsha river	98	(Yang et al. 2023)
2022–2023	Mainstream of Jinsha river	68	(Wang et al. 2024)

**TABLE 4:** Species composition in the Jinsha river mainstream based on conventional fishing and eDNA metabarcoding.

*N*	Species	Con	eDNA	*N*	Species	Con	eDNA
1	*S. sinensis*	*	*	47	*R. brunneus*	—	*
2	*S. taeniatus*	*	*	48	*X. argentea*	—	*
3	*A. rivularis*	*	*	49	*A. yunnanensis*	—	*
4	*C. alburnus*	*	*	50	*A. liui*	—	*
5	*H. leucisculus*	*	*	51	*O. elongatus*	—	*
6	*H. bleekeri*	*	*	52	*C. heterodon*	—	*
7	*H. tchangi*	*	*	53	*M. amblycephala*	—	*
8	*C. idella*	*	*	54	*P. engraulis*	—	*
9	*C. auratus*	*	*	55	*R. typus*	—	*
10	*C. carpio*	*	*	56	*M. albus*	—	*
11	*H. molitrix*	*	*	57	*G. sinensis*	—	*
12	*S. kozlovi*	*	*	58	*P. pratti*	—	*
13	*Z. platypus*	*	*	59	*P. pardalis*	—	*
14	*S. malacanthus*	*	*	60	*I. punctatus*	—	*
15	*O. bidens*	*	*	61	*L. kingi*	—	*
16	*P. parva*	*	*	62	*Onychostom sima*	*	—
17	*R. sinensis*	*	*	63	*Ancherythroculter nigrocauda*	*	—
18	*S. dabryi*	*	*	64	*Cyprinus carpio specularis*	*	—
19	*P. kaznakovi*	*	*	65	*Schizothorax wangchiachii*	*	—
20	*A. nobilis*	*	*	66	*Schizothorax prenanti*	*	—
21	*P. rabaudi*	*	*	67	*Schizothorax malacanthus*	*	—
22	*J. sinensis*	*	*	68	*Schizothorax chongi*	*	—
23	*M. anguillicaudatus*	*	*	69	*Schizothorax davidi*	*	—
24	*T. stenura*	*	*	70	*Schizothorax dolichonema*	*	—
25	*M. asiaticus*	*	*	71	*Schizothorax longibarbus*	*	—
26	*R. giurinus*	*	*	72	*Percocypris pingi*	*	—
27	*S. lucioperca*	*	*	73	*Garra pingi pingi*	*	—
28	*C. zillii*	*	*	74	*Dsicogobio yunnanensis*	*	—
29	*O. niloticus*	*	*	75	*Rhodeus lighti*	*	—
30	*O. aureus*	*	*	76	*Rhodeus ocellatus*	*	—
31	*C. gachua*	*	*	77	*Pseudogyrinocheilus prochilus*	*	—
32	*M. salmoides*	*	*	78	*Acheilognathus macropterus*	*	—
33	*P. fulvidraco*	*	*	79	*Cultrichthys erythropterus*	*	—
34	*P. vachelli*	*	*	80	*Schistura dabryi dabryi*	*	—
35	*P. anteanalis*	*	*	81	*Trilophysa bleekeri*	*	—
36	*E. kishinouyei*	*	*	82	*Trilophysa stoliczkae*	*	—
37	*S. asotus*	*	*	83	*Trilophysa huidongensis*	*	—
38	*R. lagowskii*	*	*	84	*Trilophysa anterodorsalis*	*	—
39	*A. obtusirostris*	—	*	85	*Paramisgurnus dabryanus*	*	—
40	*A. kurematsui*	—	*	86	*Paramisgurnus potanini*	*	—
41	*A. chankaensis*	—	*	87	*Neosalanx tangkahkeii*	*	—
42	*J. abbreviata*	—	*	88	*Micropercops swinhonis*	*	—
43	*S. fasciolatus*	—	*	89	*Pelteobaggrus nitidus*	*	—
44	*T. orientalis*	—	*	90	*Hystus macropterus*	*	—
45	*N. taihuensis*	—	*	91	*Silurus meridionalis*	*	—
46	*R. cliffordpopei*	—	*y				

*Note:* —indicates no detection of the species. *indicates detection of the species.

### 3.4 Fish Diversity Analysis

**FIGURE 4:**
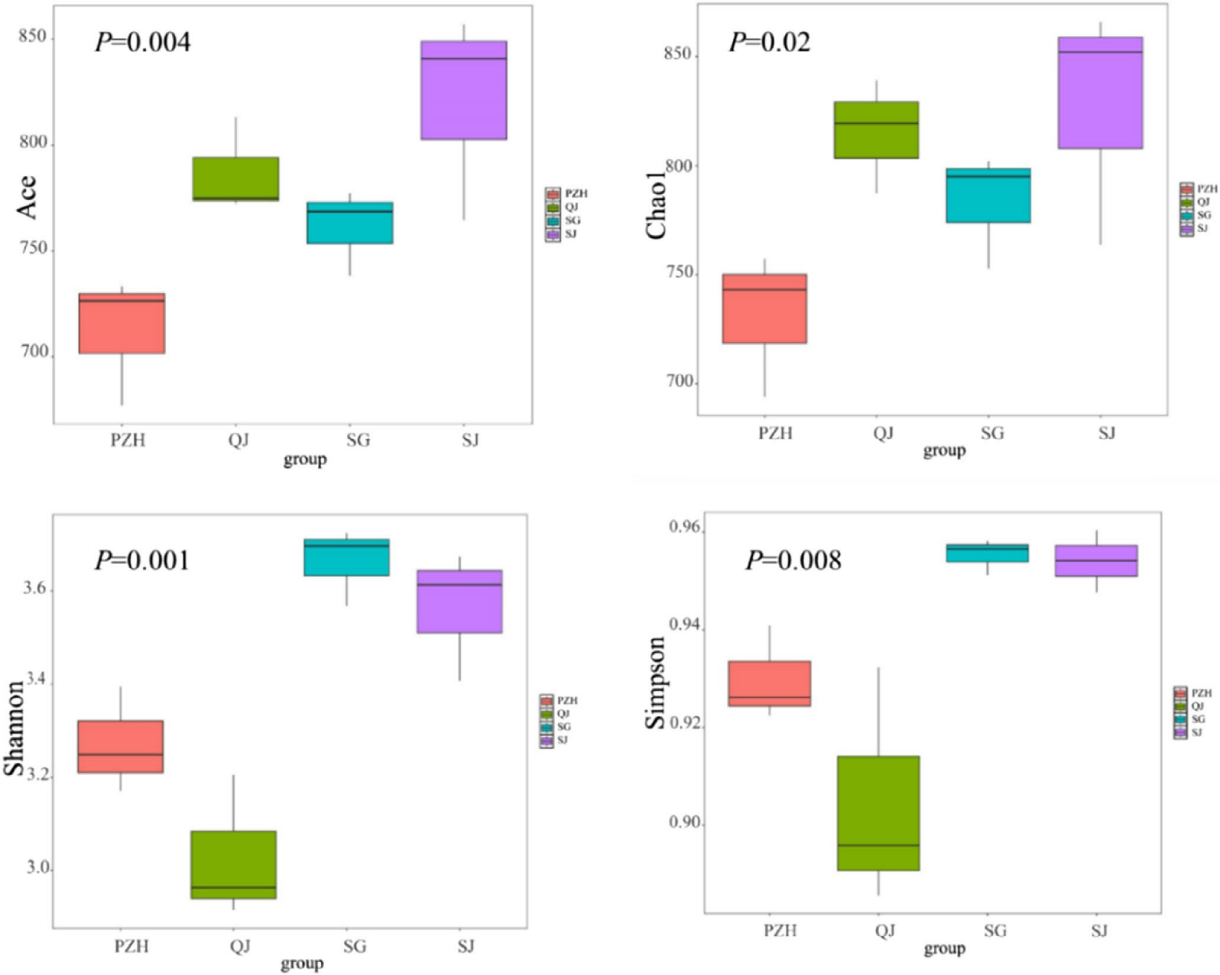
Box plot of fish alpha diversity indices at each sampling site in the Jinsha river. ANOVA analysis.

**FIGURE 5:**
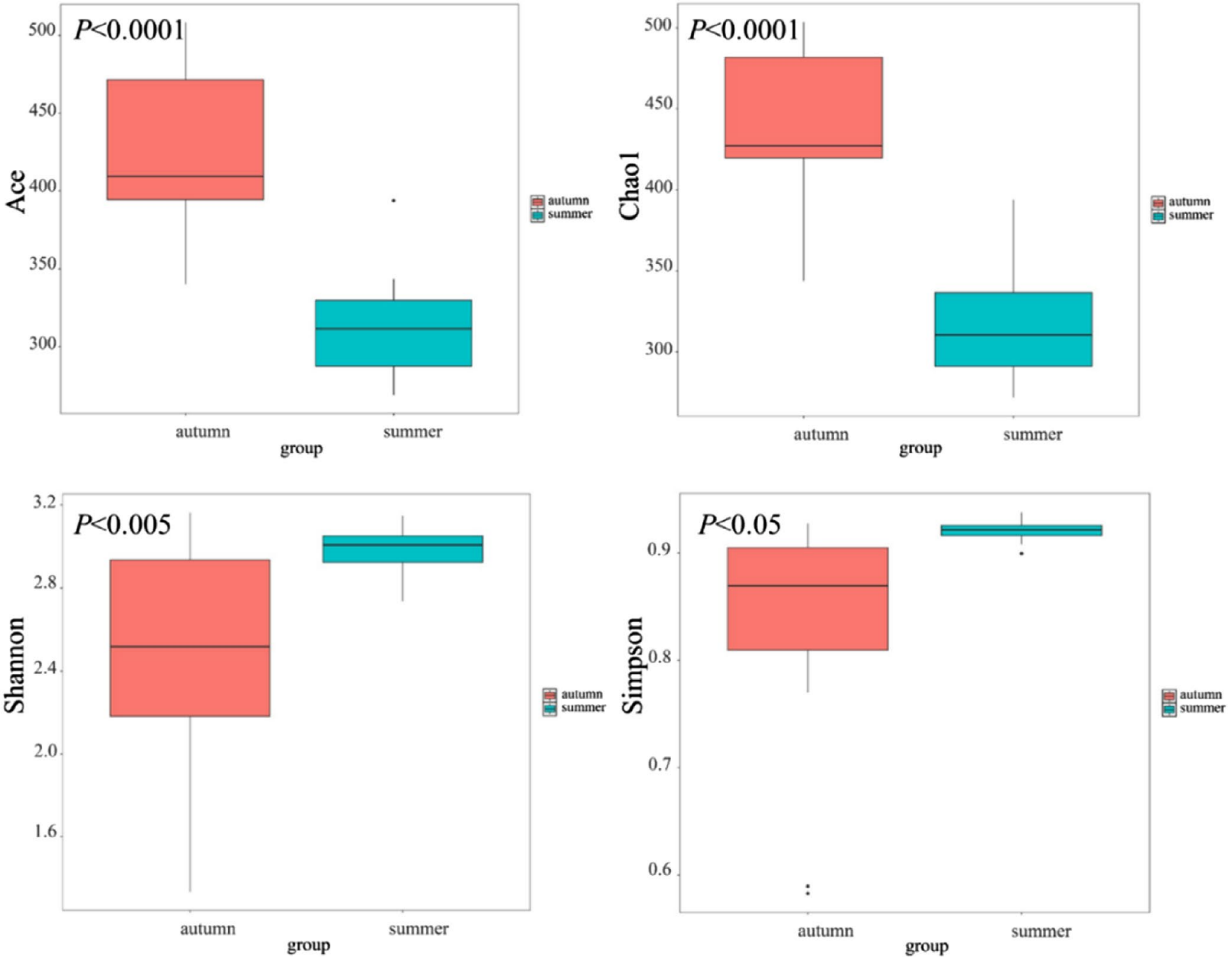
Box plot of fish alpha diversity indices across seasons in the Jinsha river. Student's *t*‐test.

The Alpha diversity indices of fish in the Jinsha river were calculated as follows: the Chao1 index ranged from 731.383 to 827.161, the ACE index from 712.245 to 820.722, the Shannon diversity index from 3.028 to 3.663, and the Simpson diversity index from 0.905 to 0.955 (Table 5). Significant differences were observed among the sites for all diversity indices. Specifically, SJ consistently exhibited the highest diversity across all indices, while PZH and QJ showed lower values (Figure 4). The Chao1 and ACE indices, which reflect species richness based on OUT abundance, were significantly higher in SJ compared to other sites. Similarly, the Shannon and Simpson indices, which incorporate both species richness and evenness, were significantly different among sites, with SG and SJ demonstrating greater diversity (Figure 4). In addition, the seasonal variations in fish alpha diversity indices between autumn and summer were shown in Figure 5. Across all indices, diversity was significantly higher in autumn than in summer. Species richness indicators, Ace and Chao1, were markedly elevated in autumn, suggesting a richer fish community during this season.

**TABLE 5:** Alpha diversity indices of fish across seasons and sampling sites in the Jinsha river.

Sampling sites	Chao1 index	Ace index	Shannon diversity index	Simpson diversity index
PZH	731.383	712.245	3.271	0.930
QJ	815.269	786.801	3.028	0.905
SG	783.316	761.390	3.663	0.955
SJ	827.161	820.722	3.565	0.954

**FIGURE 6:**
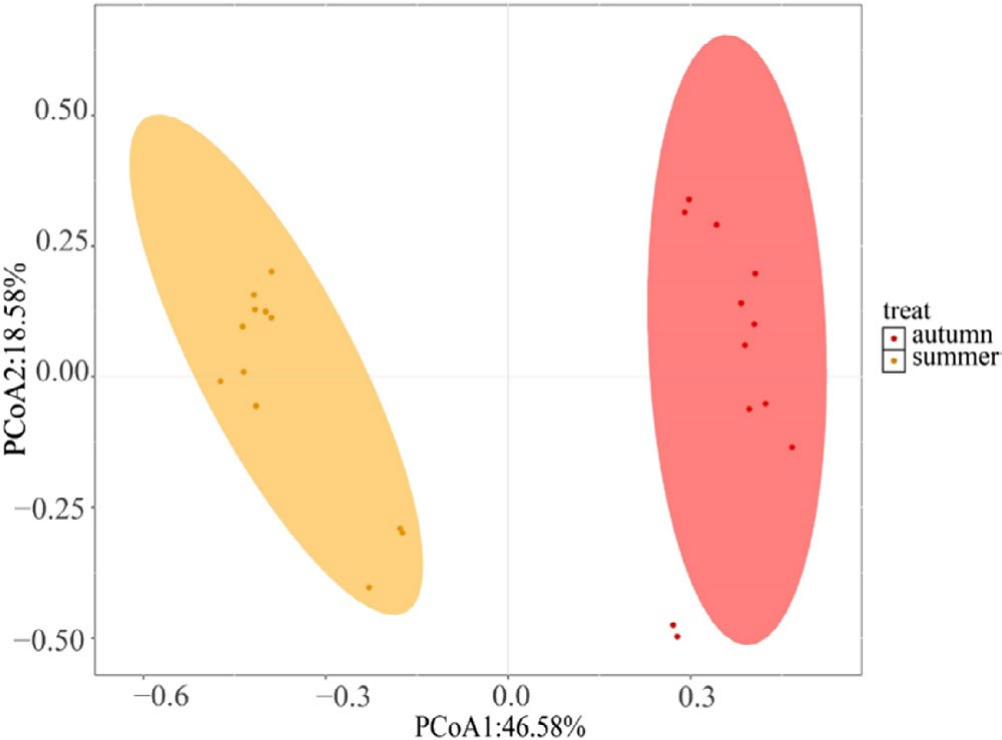
Principal coordinate analysis (PCoA) of fish community composition in the Jinsha river.

Principal Coordinate Analysis (PCoA) of species‐level sequence abundance revealed pronounced seasonal variation in fish community composition, with a clear distinction observed between summer and autumn (Figure 6). Permutational Multivariate Analysis of Variance (PERMANOVA) further confirmed this pattern, showing a significant seasonal effect on community structure (*R* ^2^ = 0.446, *p* = 0.001). These results indicated a high degree of heterogeneity and a statistically significant difference in species composition between the two seasons.

### 3.5 Relationship Between Fish Community and Environmental Factors

**FIGURE 7:**
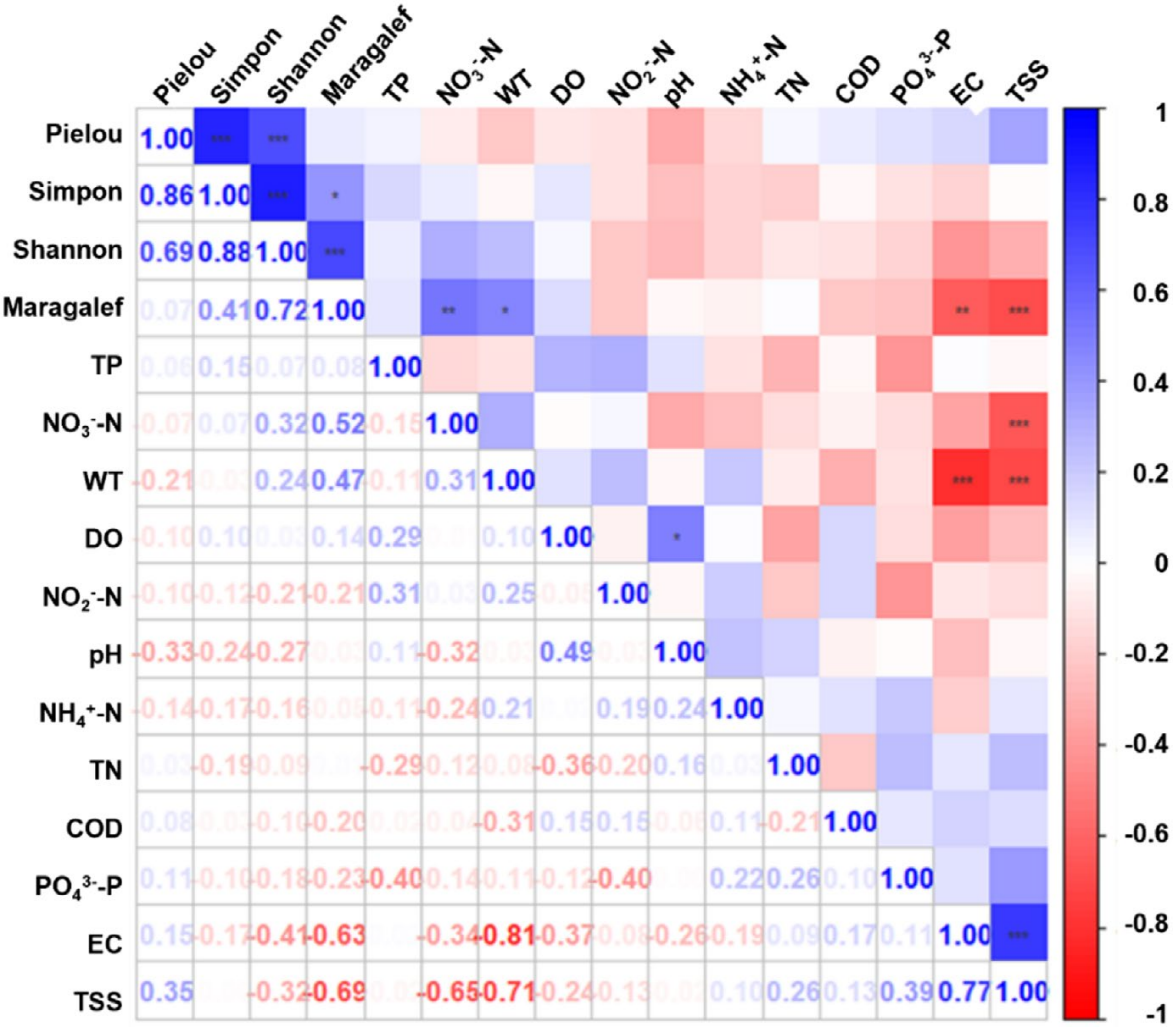
Spearman correlation analysis of environmental factors and fish alpha diversity in the Jinsha river. The legend on the right represents different *R* value intervals. *0.01 < *p* < 0.05, **0.001 < *p* < 0.01, ***0.0001 < *p* < 0.001.

We conducted Spearman correlation analysis between environmental factors and fish community Alpha diversity in the Jinsha river main stem. The Maragalef index was significantly positively correlated with NO_3_^−^—N (*R* = 0.520, *p* = 0.009) and WT (*R* = 0.469, *p* = 0.001), while negatively correlated with TSS (*R* = −0.693, *p* = 0.000) and EC (*R* = −0.629, *p* = 0.001). (Figure 7).

### 3.6 Relationship Between Fish Community Structure and Environmental Factors

**FIGURE 8:**
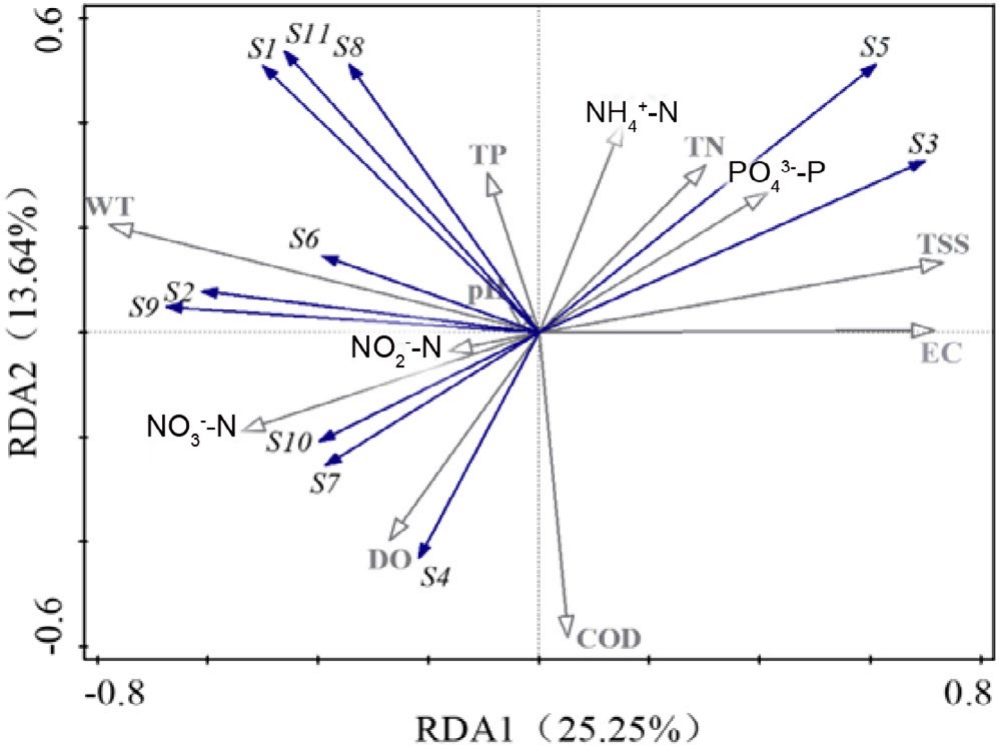
RDA ordination of fish community structure based on environmental factors and eDNA data from the Jinsha river. S1–11 correspond to the following species, respectively: *Hypophthalmichthys molitrix* , *Coptodon zillii*, *Schizothorax dolichonema* , *Rhodeus sinensis* , *Schizothorax wangchiachii* , *Hemiculter tchangi* , *Pelteobagrus vachelli*, *Aristichys nobilis*, *Carassius auratus* , *Hemiculter leucisculus* , *Cyprinus carpio* , *Rhodeus sinensis* , *Pelteobagrus vachelli*, and *Hemiculter leucisculus*.

DCA results indicated that the length of the primary ordination axis was less than 3, suggesting that Redundancy Analysis (RDA) was appropriate for this dataset. To minimize the influence of rare species, only dominant species and key environmental factors from each season and sampling site were included in the analysis. RDA, based on the eDNA survey results from the mainstem of the Jinsha river, revealed that environmental factors had a significant effect on fish community structure (*F* = 4.3, *p* = 0.006) (Figure 8). Axis I and Axis II explained 25.25% and 13.64% of the total variance, respectively. Environmental variables varied significantly across river sections and had a marked impact on the abundance of dominant fish species. Among them, WT, NO_3_^−^—N, TSS, and DO were identified as key influencing factors. The RDA results indicated that WT and NO_3_^−^—N content positively influence the abundance of *Coptodon zillii* and *Carassius auratus* . The abundance of *Pelteobagrus vachelli* and *Hemiculter leucisculus* was positively influenced by NO_3_^−^—N and DO content, while *Schizothorax dolichonema* and *Schizothorax wangchiachii* abundance was positively influenced by TN and TSS content.

## 4 Discussion

### 4.1 Overview of eDNA‐Based Fish Monitoring

This study demonstrates the effectiveness of eDNA metabarcoding in assessing fish diversity in the Jinsha river. eDNA identified 61 species, including seven invasive and several native species. Compared to conventional fishing methods (68 species), both methods shared 38 species, while eDNA uniquely detected 23 species not recorded by traditional methods (Wang et al. 2024). Several factors may explain the differences: first, eDNA is highly sensitive and can detect species through DNA traces in the environment, including those that are rare to observe. Second, some species may have cryptic behavior, small size, or inhabit areas that are hard to sample using conventional techniques. Third, eDNA reflects species presence over time and space, and DNA may come from nearby or upstream habitats, offering a broader detection scope. These results underscore the high sensitivity of eDNA for detecting both native and non‐native taxa, as well as capturing seasonal and spatial variations in community composition. The accuracy of eDNA‐based surveys is influenced by various biological and technical factors, including DNA shedding and degradation rates, which are affected by species traits, environmental conditions, and molecular properties (Stewart 2019). Additionally, primer specificity and methodological consistency are crucial for accurate species identification and reliable amplification (Dejean et al. 2011). The findings align with the growing body of literature that positions eDNA as a powerful tool for monitoring fish communities in large river systems, further validating its application in ecological research and biodiversity conservation.

### 4.2 Composition and Dominant Species of Fish Communities

Cypriniformes comprised 65.6% of all detected species, confirming their dominance in the Jinsha river, consistent with traditional fishing methods (Wang et al. 2024; Yan et al. 2022). Among the dominant species, *Ctenopharyngodon idella* , *Coptodon zillii*, *Hemiculter leucisculus* , *Aristichthys nobilis*, *Hemiculter tchangi* , *Cyprinus carpio* , *Rhinogobius cliffordpopei* , and *Neosalanx taihuensis* were the most abundant and widely distributed, with OTUs corresponding to these species detected across nearly all sampling sites. This widespread detection may reflect their high population sizes, greater biomass, and enhanced environmental adaptability. Of particular concern is the identification of seven non‐native invasive species, including *Coptodon zillii*, which was consistently observed at high relative abundance. *Coptodon zillii has become a dominant species in the Jiulong river Basin of Southeast China, posing significant ecological risks to the native fish community* (Feng et al. 2025). The presence of such species highlights ongoing ecological invasion pressures in the Jinsha river and underscores the need for early‐warning monitoring strategies based on eDNA.

### 4.3 Temporal and Spatial Patterns in Fish Diversity

PCoA revealed significant seasonal differences in fish community composition, with 25 species common to both summer and autumn, and more species detected in autumn. In contrast, eDNA metabarcoding of the Chongqing section of the upper Yangtze river indicated seasonal variations in fish composition, with the richness index being higher in summer compared to other seasons (Shen et al. 2023). These differences likely reflect environmental influences on eDNA distribution, such as changes in water temperature, flow velocity, and turbidity. Additionally, fish behavior—including reproductive activity, seasonal migration, and overwintering, may also affect the eDNA shedding rate, thereby contributing to seasonal patterns in species detectability. Furthermore, spatial variation in diversity was evident across sampling sites. Fluctuations in fish species detection and eDNA relative sequence abundance at various sampling locations may indicate changes in habitat utilization and distribution (Stoeckle et al. 2017). Notably, the SJ site, located in a sheltered cove, exhibited higher Chao1 and ACE index values. This may be attributed to the relatively stagnant water conditions and the input of bait from recreational fishing, which together promote local fish aggregation and increase the likelihood of eDNA detection. In contrast, sites with stronger currents may experience faster eDNA dispersion or dilution, reducing the likelihood of local detection.

### 4.4 Environmental Drivers of Community Structure

Statistical analyses revealed significant correlations between fish diversity indices and environmental variables, particularly WT and DO, NO_3_^−^—N and TSS. RDA further indicated that WT was a key factor influencing community structure, which aligns with findings from previous studies (Cao et al. 2025; Lai et al. 2024; Qian et al. 2023). In this study, *Coptodon zillii*, *Hemiculter tchangi* , and *Carassius auratus* were associated with higher WT, whereas *Schizothorax dolichonema* and *Schizothorax wangchiachii* preferred lower WT; this pattern is closely linked to the regulatory role of WT in shaping fish life‐history traits. As WT of river increases, DO levels tend to decline, and excessively low DO can impose physiological stress on fish. For example, low DO has been shown to reduce species richness and alter community composition, whereas maintaining adequate DO levels is essential for sustaining fish populations and preserving ecosystem health, as highlighted in the Pearl river Estuary study (Lai et al. 2024). In the present study, species such as *Rhodeus sinensis* , *Pelteobagrus vachelli*, and *Hemiculter leucisculus* exhibited strong positive correlations with DO levels, suggesting their preference for well‐oxygenated environments. Additionally, seasonal decreases in DO, often associated with elevated summer temperatures, may further exacerbate stress in sensitive species.

Moreover, elevated levels of NO_3_^−^—N and TSS are widely recognized as key indicators of anthropogenic pollution in aquatic ecosystems. Species such as *Hypophthalmichthys molitrix* , *Coptodon zillii*, *Rhodeus sinensis* , and *Aristichys nobilis* were predominantly found in warm, well‐oxygenated waters with low nutrient concentrations, indicating a preference for clean water environments. In contrast, species including *Schizothorax dolichonema* , *Schizothorax wangchiachii* , *Hemiculter tchangi* , *Carassius auratus* , *Hemiculter leucisculus* , and *Cyprinus carpio* were commonly observed in polluted waters, exhibiting tolerance to high levels of organic matter, elevated nitrogen and phosphorus concentrations, and increased turbidity. These species may serve as potential indicators of eutrophication. These species‐specific reactions to environmental variables highlight the complex ecological dynamics in the Jinsha river and contribute to the observed heterogeneity in fish community structure.

Our findings align with eDNA‐based studies in other ecosystems. For example, Dong et al. identified DO, water level, and flow velocity as key factors shaping fish communities in freshwater lakes and upstream rivers (Dong et al. 2023). Furthermore, watershed‐scale variables such as elevation, river width, and surrounding land cover also influence fish community structure. While this study focused on in‐stream environmental parameters, future research should incorporate broader landscape‐level variables to gain a more comprehensive understanding of the multi‐scalar drivers of fish diversity.

### 4.5 Implications and Future Research

This study demonstrates the utility of eDNA metabarcoding as a powerful tool for assessing biodiversity in large river systems. The fish species richness and abundance detected in this study were found to closely align with those observed using traditional survey methods, indicating its ability to detect a wide range of native and invasive species, capture seasonal patterns, and quantify environmental correlations underscores its potential for ecological monitoring and resource management in the Jinsha river Basin during the “Ten‐Year Fishing Ban” period. The fishing ban has the potential to facilitate the recovery of native species by alleviating fishing pressure and providing increased opportunities for reproduction. This, in turn, could contribute to the restoration of ecological balance and enhance species diversity. However, the ban may have variable effects on invasive species. While it could reduce the competitive pressure on native species from invasives, it may also create favorable conditions for the proliferation of invasive species if not adequately managed. Ultimately, the long‐term success of the fishing ban will depend on its duration, the scope of its implementation, and the effectiveness of supplementary management strategies aimed at controlling invasive species and promoting ecosystem recovery.

Future research should focus on integrating eDNA monitoring with traditional survey methods and hydrological modeling to enhance spatial accuracy and ecological inference. Additionally, incorporating watershed‐scale environmental and land‐use variables will yield a more comprehensive understanding of the factors driving biodiversity in the Jinsha river and beyond. Such integrative approaches are crucial for supporting long‐term conservation efforts and adaptive management strategies in dynamic riverine ecosystems.

## Author Contributions

**Yan Zhao:** conceptualization (lead), data curation (lead), methodology (lead), software (equal), supervision (equal), writing – original draft (equal), writing – review and editing (equal). **Zhongyuan Wang:** conceptualization (equal), data curation (equal), formal analysis (equal), investigation (equal), methodology (equal), software (equal), supervision (equal), validation (equal), writing – original draft (equal). **Feifei Hu:** data curation (equal), formal analysis (equal), investigation (equal), methodology (equal). **Zhibin Guo:** software (equal), supervision (equal), validation (equal). **Jinling Gong:** software (equal), supervision (equal). **Xuemei Li:** funding acquisition (equal), writing – original draft (equal), writing – review and editing (equal). **Deguo Yang:** software (equal), validation (equal). **Tingbing Zhu:** conceptualization (equal), funding acquisition (equal), writing – review and editing (equal).

## Ethics Statement

All experimental procedures were carried out in accordance with the guidelines for the ethical review of animal experiments at the Experimental Animal Center of the Yangtze river Fisheries Research Institute, Chinese Academy of Fishery Sciences (ID Number: 2022YFI‐ZTB‐02).

## Conflicts of Interest

The authors declare no conflicts of interest.

## Supporting information


**Data S1:** ece371997‐sup‐0001‐Supinfo.pdf.

## Data Availability

The data sets supporting the results of this article are available in the NCBI SRA repository, accession number PRJNA1283050. All the required data are uploaded as Supporting Information.
