# Supplementary material for: Fish Diversity and Environmental Relationships in the Jinsha River During the Initial Phases of the 10‐Year Fishing Ban: A Metabarcoding Approach
Source: Ecol Evol. 2025 Aug 14;15(8):e72002. doi: 10.1002/ece3.72002 (PMC12351800; doi:10.1002/ece3.72002)
Supplement: Supplementary file 1 — Data S1: ece371997‐sup‐0001‐Supinfo.pdf. [file ECE3-15-e72002-s001.pdf]

eDNA reveals spatiotemporal fish diversity patterns amid Yangtze fishing ban.

eDNA metabarcoding effectively detects diverse native and invasive fish taxa.

Cypriniformes dominate; invasive species highlight ecological vulnerability.

Fish diversity varies spatiotemporally, shaped by environment and behavior.

WT, DO, and pollutants significantly structure fish community composition.

For Review Only

		PO4-P	NO3-N	NO2-N	NH4-N	TN	TP	TSS
May-22	SG	0.35	0.3	0.004	0.6	1	0.14	36
	PZH	0.05	0.3	0.004	0.02	0.4	0.15	16
	QJ	0.02	0.6	0.006	0.04	0.5	0.3	3
	SJ	0.01	0.4	0.005	0.02	0.5	0.29	10
	BZL	0.08	0.2	0.002	0.29	1.5	0.07	58
	GT	0.08	0.3	0.006	0.02	25	0.12	140
Oct-22	SG	0.06	0.5	0.003	0.21	1	0.06	93
	PZH	0.05	0.6	0.006	0.05	0.56	0.05	7
	QJ	0.44	0.8	0.003	0.02	15.7	0.11	12
	SJ	0.1	0.8	0.004	0.02	0.3	0.09	7
	BZL	0.64	0.5	0.003	0.07	0.5	0.39	52
	GT	0.16	0.5	0.005	0.01	0.6	0.26	158
May-23	SG	0.03	0.9	0.004	0.02	1.5	0.07	15
	PZH	0.03	0.5	0.004	0.02	0.9	0.15	5
	QJ	0.01	0.5	0.006	0.28	1.5	0.50	8
	SJ	0.00	0.7	0.004	0.00	0.7	0.25	2
	BZL	0.03	0.5	0.003	0.01	0.2	0.15	16
	GT	0.03	0.4	0.005	0.02	0.9	0.24	182
Oct-23	SG	0.03	0.5	0.003	0.01	1.2	0.7	33
	PZH	0.01	0.8	0.006	0.02	0.3	0.17	6
	QJ	0.06	0.7	0.003	0.01	1	0.09	4
	SJ	0.07	0.7	0.003	0.01	2.7	0.08	4
	BZL	0.04	0.4	0.003	0.01	0.8	0.11	82
	GT	0.05	0.4	0.004	0.01	3	0.14	182

For Review Only

COD	WT	pH	DO	σ
11	18.1	8.24	8.11	571
8	23	8.14	8.73	584
7	22.6	8.45	9.7	471
11	18.1	8.91	10.37	480
5	17.4	8.59	8.6	677
3	18.9	8.86	7.89	1121
9	14.7	8.35	8.43	1044
38	21.2	8.31	8.4	618
1	21.6	8.02	7.94	494
21	22.7	8.35	9.01	404
9	12.1	8.71	9.2	1175
22	7.6	7.78	8.57	2124
15	13.9	7.97	8.26	1262
22	17.4	8.56	8.39	521
3	24.4	8.36	8.57	507
4	19.8	8.18	8.87	535
5	15.9	7.5	7.94	1303
10	13	7.8	7.21	1842
14	12.7	8.41	8.58	1096
2	19.4	8.32	8.33	617
3	22	8.27	7.45	517
7	22.7	8.4	8.78	503
3	11.8	8.47	8.81	1100
9	7.6	8.37	8.41	1601

For Review Only

	M1	M2	M3	M4	M5	M6	M7	M8
SG	0.012319	0	0.016031	0.029352	0	0.00	0	0.015611
PZH	0.135818	0.091885	0.042449	0.072277	0.157494	0.00	0	0.041175
QJ	0.04671	0.294901	0.136064	0.292614	0.148656	0.10	0.039129	0
SJ	0.472628	0.1697	0.22695	0.014501	0	0.00	0.035802	0.012786
SG	0	0.280123	0	0.153529	0.180196	0.57	0	0.043677
PZH	0	0.511168	0	0.064455	0	0.65	0	0.368976
QJ	0	0.007396	0.012628	0	0.006805	0.29	0.006528	0.035155
SJ	0.02041	0.040039	0.019153	0.128914	0.023626	0.59	0.019833	0.098468

For Review Only

M9
0
0
0.026605
0
0.073096
0.018446
0
0.037229

For Review Only
